# Reemergence of Human and Animal Brucellosis, Bulgaria

**DOI:** 10.3201/eid1502.081025

**Published:** 2009-02

**Authors:** Gianluca Russo, Paolo Pasquali, Roumiana Nenova, Tsviatko Alexandrov, Stanislav Ralchev, Vincenzo Vullo, Giovanni Rezza, Todor Kantardjiev

**Affiliations:** Sapienza University, Rome, Italy (G. Russo, V. Vullo); Istituto Superiore di Sanità, Rome (P. Pasquali, G. Rezza); National Veterinary Medical Service, Sofia, Bulgaria (T. Alexandrov, S. Ralchev); National Center for Infectious and Parasitic Diseases, Sofia (R. Nenova, T. Kantardjiev); 1 These authors equally contributed to this article.

**Keywords:** Brucellosis, Bulgaria, zoonosis, infection reemergence, dispatch

## Abstract

Bulgaria had been free from brucellosis since 1958, but during 2005–2007, a reemergence of human and animal disease was recorded. The reemergence of this zoonosis in the country highlights the importance of maintaining an active surveillance system for infectious diseases that will require full cooperation between public health and veterinary authorities.

According to the World Health Organization (*1*), brucellosis is one of the most common zoonoses worldwide and is considered a reemerging infectious disease in many areas of the world. An estimated 500,000 new human cases occur annually worldwide (*2*). In Europe, 1,033 human brucellosis cases were reported in 2006 (*3*); data from a passive surveillance system were based on clinical findings, supported by epidemiologic criteria, and confirmed by serologic tests. Here we report the results of a survey performed in Bulgaria during 2005–2007, which has been considered free from *Brucellosis melitensis* and *B. abortus* disease since 1958 (*4*).

In Bulgaria, until 1998 serologic screening was mandatory for all cattle, sheep, and goats >12 months of age. Afterward, based on risk assessment, animal surveillance activities covered 100% of heads reared in municipalities along the borders with countries endemic for brucellosis such as Turkey, Greece, and the former Yugoslav Republic of Macedonia; 50% of the animals reared in other municipalities of the regions bordering the aforementioned countries; and 25% of animals reared in the inner Bulgarian regions. Currently, an active surveillance system is in place for dairy factory employees and persons considered at risk after outbreaks in ruminants.

## The Study

**Figure 1:**
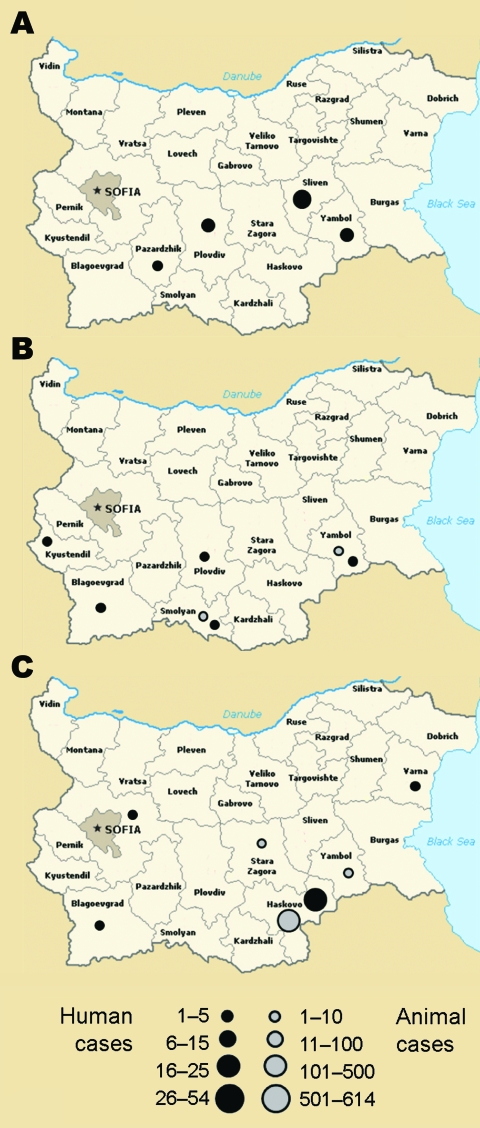
Geographic distribution of human and animal brucellosis in Bulgaria during A) 2005, B) 2006, and C) 2007.

During 2005–2007 (Figure 1), a total of 105 human cases of brucellosis were diagnosed among 2,054 persons who were tested on the basis of clinical suspicion or risky exposure. A human case of brucellosis was considered confirmed if results of serologic tests, such as ELISA or complement fixation test, were positive, in accordance with the World Health Organization case definition (*5*). Bacteria isolation and characterization had not been performed routinely.

The alert started in 2005 (Figure 1, panel A), when a case of brucellosis occurred in a Bulgarian migrant animal keeper working in Greece. Active surveillance of persons at risk was implemented, enabling detection of a total of 34 human cases of brucellosis. All cases were classified as imported cases; therefore, no supplemental active surveillance on animals was implemented. Additionally, during routine screening for at-risk workers, 3 other persons employed in a dairy factory were found to be seropositive. Due to the lack of traceability of the raw material used in the factory, it was not possible to trace the origin of the infection. At that time, there was no evidence of animal cases of brucellosis.

During 2006 (Figure 1, panel B), a total of 10 cases of human brucellosis were reported from different regions of the country. According to anamnestic information, these case-patients had different sources of infection: 3 of the 10 were considered imported infections; 1 case-patient was diagnosed during hospitalization in Sicily (Italy), where the patient reported having eaten ricotta cheese, and 2 occurred in Bulgarian migrant animal keepers working in Greece. Concerning the origin of infection, epidemiologic data suggest that 5 of the 10 cases were related to occupational risk and the remaining to consumption of raw milk and milk derivates. Surveillance activities enabled detection of 10 animals (7 small ruminants and 3 cows) with positive serologic results; these animals were then killed and destroyed. During 2007 (Figure 1, panel C), a total of 58 human cases were identified. Of 58 cases, 54 were classified as autochthonous (i.e., acquired by imported animals found to be infected during regular veterinary surveillance). These cases were identified in a Bulgarian region bordering Greece and Turkey (Haskovo region).

Two other cases, which were also classified as autochthonous, were diagnosed in patients who stated they had consumed a risky product (i.e., raw milk handled without adherence to hygienic standards). The remaining 2 cases were classified as imported because they involved Bulgarian migrant animal keepers working in Greece. Active surveillance in place for animals found a total of 625 heads (618 small ruminants, 7 cows) with positive serologic results; all were killed and destroyed. Analagous with what we observed in humans, most of the infected animals were found in the Haskovo region. All animals found to be infected during surveillance activity were bred at the family farm, and their milk and dairy products were prepared and eaten without adherence to proper hygienic standards.

## Conclusions

**Figure 2:**
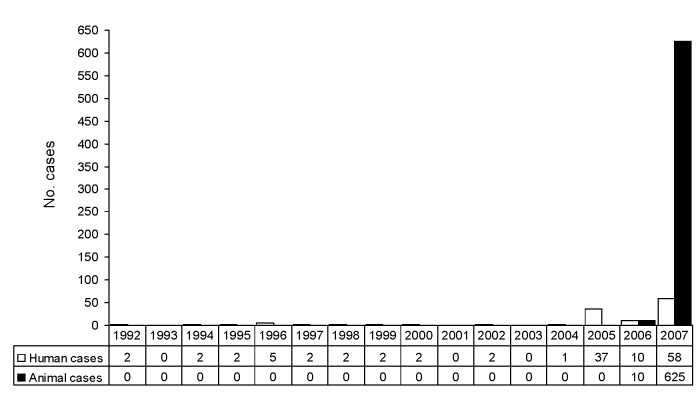
Human and animal cases of brucellosis in Bulgaria, 1992–2007. In Bulgaria, during 1992–2004, a total of 22 human cases and 0 animal cases of brucellosis were recorded; during 2005–2007, a total of 105 human cases and 635 animal cases of brucellosis were recorded.

Our data show that brucellosis is reemerging in Bulgaria (Figure 2). On the basis of information provided in this report, we can make several hypotheses regarding the causes of the resurgence of a previously controlled infection in a transitional, rapidly changing country.

Overall, 105 human cases of human brucellosis were identified over a 3-year period. Of them, 84 cases (80%) were identified in persons at occupational risk. This finding suggests that when brucellosis is introduced into naive territories (i.e., those territories that were considered officially free of brucellosis), the primary source of infection for humans is direct contact with infected animals (i.e., exposure to abortion/delivery products) or domestic consumption of products produced on family farms (milk, raw cheese). However, environmental exposure can also occur, especially in infants and children, who are considered at lower risk for direct contact with potentially infected animals, as recently observed (*6*). This hypothesis appears to be consistent with the context of a naive setting, where preventive measures are not routinely implemented. Continuous health education and other strategies may contribute to reduce the circulation of human brucellosis in endemic areas (*7*).

The reemergence of brucellosis is not limited to Bulgaria but involves several countries in the Balkan region and even in the Caucasian region (P. Pasquali, unpub. data). This trend or reemergence has several explanations. First, due to socioeconomic changes, many countries in these regions are experiencing a dramatic increase of animal trade, animal movement, and occupational migration, which in turn may increase the risk for introduction and spread of infectious diseases, such as brucellosis, from other disease-endemic countries like Greece or Turkey (*2*). Second, the process that has characterized the change of the social and administrative organization since the collapse of the Soviet Union is far from being completed; the public health systems are still flawed in many countries. Finally, part of the increase may simply be that brucellosis is a complex disease, which has different cycles of expansion and regression.

Before drawing conclusions, we should mention 2 possible limitations of the study. First, samples from patients with positive serologic results were used for bacterial culture for brucellosis only if sample collection was properly timed; no culture positive case is available. Second, we cannot exclude the possibility that part of the increase in cases of brucellosis could be due to improved surveillance; in particular, temporal trends and geographic comparison might be, to some extent, affected by the intensity of screening activities. However, this increased surveillance is unlikely to bias the observed shift from imported to locally acquired cases.

In conclusion, this report shows how a disease such as brucellosis may increase its public health impact, particularly in transitional countries such as Bulgaria. Our findings emphasize the importance of the combination of health education and active surveillance systems for controlling infectious diseases and highlight the need for cooperation between public health officials and veterinary officers. Creating and improving capacity building are necessary to properly address issues that pose public health hazards.
